# Aspiration in injections: should we continue or abandon the practice?

**DOI:** 10.12688/f1000research.1113.3

**Published:** 2017-03-01

**Authors:** Yasir Sepah, Lubna Samad, Arshad Altaf, Muhammad Sohail Halim, Nithya Rajagopalan, Aamir Javed Khan

**Affiliations:** 1 Centre for Injection Safety Pakistan, Interactive Research & Development, Karachi, Pakistan; 2 Stanford University, Stanford, CA, USA; 3 The Indus Hospital, Karachi, 75190, Pakistan; 4 Bloomberg School of Public Health, Johns Hopkins University, Baltimore, MD, USA; 5 Canada-Pakistan HIV/AIDS Surveillance Project, Sindh AIDS Control Programme, Karachi, Pakistan; 6 University of Nebraska Medical Center, Omaha, NE, USA

**Keywords:** Aspiration, injection

## Abstract

Aspiration during any kind of injection is meant to ensure that the needle tip is at the desired location during this blind procedure. While aspiration appears to be a simple procedure, it has generated a lot of controversy concerning the perceived benefits and indications. Advocates and opponents of aspiration both make logically sound claims. However, due to scarcity of available data, there is no evidence that this procedure is truly beneficial or unwarranted. Keeping in view the huge number of injections given worldwide, it is important that we draw attention to key questions regarding aspiration that, up till now, remain unanswered. In this review, we have attempted to gather and present literature on aspiration both from published and non-published sources in order to provide not only an exhaustive review of the subject, but also a starting point for further studies on more specific areas requiring clarification. A literature review was conducted using the US National Institute of Health’s PubMed service (including Medline), Google Scholar and Scopus. Guidelines provided by the World Health Organization, Safe Injection Global Network, International Council of Nursing, Center for Disease Control, US Federal Drug Agency, UK National Health Services, British Medical Association, Europe Nursing and Midwifery Council, Public Health Agency Canada, Pakistan Medical Association and International Organization of Standardization recommendations 7886 parts 1-4 for sterile hypodermics were reviewed for relevant information. In addition, curricula of several medical/nursing schools from India, Nigeria and Pakistan, the US pharmacopeia Data from the WHO Program for International Drug Monitoring network in regard to adverse events as a result of not aspirating prior to injection delivery were reviewed. Curricula of selected major medical/nursing schools in India, Nigeria and Pakistan, national therapeutic formularies, product inserts of most commonly used drugs and other possible sources of information regarding aspiration and injections were consulted as well.

## Introduction

An injection is defined by the World Health Organization (WHO) as parenteral administration of medication through a skin puncture via a syringe, while aspiration is defined as the pulling back of the plunger of a syringe (for 5–10 seconds) prior to injecting medicine ^1– 4^. Aspiration is most commonly performed during an intramuscular (IM) or subcutaneous (SC) injection, and is meant to ensure that the needle tip is located at the desired site, and has not accidentally punctured a blood vessel.

Despite the growing wealth of medical knowledge in recent decades, the simple procedure of aspiration is still generating much controversy concerning its perceived benefits and indications ^5^. Advocates of aspiration contend that it is a technically easy maneuver that is rapidly performed and well tolerated by patients with no increase in costs incurred. However, due to a paucity of available data, there is no evidence that this procedure is essential or truly beneficial. This issue has been widely debated with specific regard to vaccination; there are no studies that have assessed the need for aspiration prior to IM injection of vaccines in relation to vaccine safety. The widespread use of auto-disable (AD) syringes – most of which are not designed to aspirate ^6^ – has not been linked to adverse effects due to the elimination of the aspiration procedure prior to injection of vaccines ^7^. This finding has intensified the debate and raised doubts over the necessity of aspiration in non-vaccine medication administration as well.

Conventional syringes are also used to aspirate materials other than blood – synovial fluid, amniotic fluid, cells (via fine needle cytology), pericardial fluid, peritoneal fluid and cerebrospinal fluid (CSF) are examples ^8– 19^. This wide spectrum of applications for conventional syringes is all the more interesting in view of the fact that although used for both aspiration and injection, the syringe is actually designed only for injection ^20^. A number of studies have concluded that a conventional syringe is a poorly controlled and non-ergonomic device during aspiration ^21, 22^. Possible lack of precision may result in local trauma and pain, prolonged procedure time, failed or incomplete procedures, accidental puncture of blood vessels or nerve bundles, poor sample retrieval and delayed diagnosis ^23– 33^. The ingrained use of the conventional syringe for injection and aspiration is to a large extent attributable to its low cost, widespread availability and lack of an effective alternative ^21^.

The huge volume of injections being given worldwide – an estimated 16 billion injections per year are administered in the developing and transitional countries alone ^34^ – necessitates that this aspect of injection technique be given due attention. This review aims to collate English-language literature on aspiration from all published and non-published sources in order to provide an overview on the subject. In particular, this review aims to highlight areas of debate and draw attention to key questions that remain unanswered, thus providing a starting point for controlled studies on specific areas requiring clarification.

## Methodology

A literature review was conducted using the US National Institute of Health’s PubMed service (including Medline), archives of SIGNpost, the weekly electronic newsletter of the World Health Organization’s (WHO) Safe Injection Global Network (SIGN), and International Organization of Standardization (ISO) recommendations 7886 parts 1–4 for sterile hypodermics. Clarification on points of debate was sought by direct communication with ISO. Google Scholar was also used to search for relevant information. Relevant search terms for PubMed and Google Scholar literature searches are listed below.

Guidelines from the WHO, International Council of Nursing (ICN), US Center for Disease Control (CDC), US Federal Drug Agency (FDA), UK National Health Service (NHS), British Medical Association, UK Nursing and Midwifery Council (NMC), and Australian Nursing and Midwifery Accreditation Council, Public Health Agency Canada and the Pakistan Medical Association (PMA) were extensively searched for information. Data from the WHO Program for International Drug Monitoring network in regard to adverse events as a result of not aspirating prior to injection delivery were reviewed. Curricula of selected major medical/nursing schools in India, Nigeria and Pakistan were also reviewed for relevant information to document the inclusion (or otherwise) of aspiration in teaching guidelines for injection technique.

National therapeutic manuals and formularies such as British National Formulary (BNF), European Pharmacopeia (EP), United States Pharmacopeia (USP) and Pakistan Pharma Guide (PPG) were also consulted for information regarding aspiration before injection. Product inserts for all injectable drugs on the WHO Essential Drug List (EDL) were collected to determine if the manufacturer had provided instructions on aspiration prior to injecting the drug. These product inserts were collected from local pharmacies and the international manufacturers for each drug. Drug inserts from multi-nationals were acquired either directly from their websites or from other online resources including the Drug Index ( www.Rxlist.com), Australian Prescription Products Guide ( www.appgonline.com.au/default.asp) and from ( http://www.rxmed.com/).

## Results

Our review was conducted between March 2008 and March 2014. Table 1 summarizes the resources searched.

**Table 1.** Description of peer items reviewed with numbers reviewed.

Items	Number of Items Reviewed	Description
Articles	78	PubMed Google Scholar
Non-indexed publications	90	Websites of WHO, CDC, ICN, Canadian Public Health Agency, UNICEF, pharmaceutical companies SIGN-Post Internet Drug Index Australian Prescription Products Guide Rxmed.com Wikipedia
International guidelines	4	WHO ICN NMC Europe ISO 7886 (part 1–4)
National Guidelines	5	NHS UK NMC UK CDC USA FDA USA PHA Canada
Medical and Nursing Curriculum	4	Aga Khan University School of Medicine, Pakistan National Nigerian Nursing School INCLEN
Formularies	3	BNF UK European Pharmacopeia US Pharmacopeia Pakistan Pharma guide
WHO Essential Drug List Product Inserts for Injectable Drugs	155 Injectable listed 104 reviewed

### Literature review findings

Published literature on injection technique advises aspiration before injecting a drug through different routes, i.e. IM ^35^, intravascular (IV) ^36^ or SC ^37^. However, it is important to note that emphasis has been placed on negative pressure being applied for 5–10 seconds for aspiration to be of benefit ^1, 3, 4^. During the administration of an IV injection, the presence of “flashback” (return of blood into the syringe or cannula) is a passive process and active aspiration is usually not necessitated; hence, this particular route of administration has not been emphasized in the review below.

***IM injections:*** Aspiration prior to injection of medication through the IM route remains a part of most guidelines ^4, 35, 38– 40^. Nursing curricula and guidelines ^4, 38, 39^ clearly recommend aspiration as an essential step in IM injection technique. Guidelines originating in the UK recommend aspiration prior to IM injection of medications ^35^, as well as specifically as part of the Z-track technique of administering IM injections. Training curricula for community health workers in Nigeria recommend aspiration prior to IM, SC and intradermal [ID] injections ^40^.

***SC injections:*** It is apparent that there are opposing schools of thought when it comes to aspiration prior to SC injections. There are those that insist that aspiration should continue to be part of SC injection techniques for medication administration, and those who are convinced that aspiration is not necessary and has no real advantage; in fact, several disadvantages may be attributed to this step.

Some nursing curricula do not include aspiration as part of the recommended technique ^38^ for SC injection. One nursing guideline highlights the debate existing over aspiration prior to a SC injection, concluding that while the likelihood of piercing a vessel is slim, local guidelines should be followed in determining individual practices. Others recommend routine aspiration prior to injection of medications through the SC route ^42^.

The WHO/ICN ^43^ combined guidelines do not mention aspiration. Similarly, the WHO/SIGN document ^44^ “A Guide For Supervising Injections” makes no recommendations related to aspiration. Both documents are primarily concerned with infection control practices in relation to injection administration, overlooking aspiration entirely.

A recent debate in relation to SC injection of immunotherapy has highlighted this controversy. Waibel recommended that aspiration before SC injection of immunotherapy be abandoned since there were no positive aspirates in 36,000 immunotherapy injections given at his practice ^45^. While other specialists agreed that aspiration prior to immunotherapy injection in SC tissue is very rarely positive, rare anecdotes were quoted when positive aspiration has been documented ^46, 47^, even in the hands of experienced specialists and nurses. Given the potentially fatal adverse reactions of immunotherapy injected into blood vessels, it is logical to recommend that aspiration be performed as part of the standard technique. However, fatal and near fatal adverse reactions have been reported following immunotherapy injection despite precautions, including aspiration, being taken ^45^.

***Epinephrine:*** Epinephrine is given through the SC or IM route to treat allergic reactions. Geller ^48^ has reported the observation of a positive aspiration prior to epinephrine injection for asthma; if aspiration had not been performed in that instance, epinephrine would have been injected into the blood vessel with potentially hazardous consequences. On the other hand, the preloaded auto injector commonly used for administering epinephrine in emergency situations does not allow for aspiration ^49^. In this form, epinephrine is designed to be administered via IV injection, via intracardiac injection or via the endotracheal route into the bronchial tree where aspiration is superfluous.

***Insulin:*** The NMC guidelines ^50, 51^ do not mention aspiration in relation to insulin injection. Aspiration prior to insulin injection is rarely positive ^36^ and hence not indicated. This recommendation is supported by drawing a parallel with heparin administration, where increased hematoma formation has been associated with aspiration ^4^.

***Dental procedures:*** A study looking at dental anesthetic injections showed positive aspiration rates ranging from 3.2–8% depending on the type of syringe system used ^52^ and the type of nerve block. Accuracy of needle position combined with mechanical ease at the time of dental injections are important considerations when choosing an appropriate device ^53^. To this end, different self-aspirating devices have been tested in dental practice ^54^. An understanding of vascular anatomy ^57^ is all the more important in view of the potential toxicity of anesthetic agents and the possibility of embolization to the ophthalmic artery ^58^.

The US CDC screening form ^55^ for device specifics notes whether a dental syringe is capable of aspiration.

***Immunization:*** Vaccinations form an important subset of all injections given worldwide. Most government programs worldwide follow UNICEF/WHO recommendation in their Expanded Program on Immunization (EPI) programs. At present, the WHO does not recommend aspiration prior to administering a vaccine ^7, 56^. Current guidelines published by the American Academy of Pediatrics (AAP) ^57^ recommend that aspiration prior to IM vaccinations may not be necessary, while similar Canadian guidelines continue to recommend aspiration ^58^. The US Advisory Committee on Immunization Practices (ACIP) ^59^ does not make any recommendations on aspiration at the time of vaccine administration. Without data indicating the need for aspiration during vaccination, ACIP is basically leaving this decision to the person giving the vaccine. A similar stance is taken by the US Immunization Action Coalition guideline ^40^ where aspiration is not mentioned in its recommendations for SC and IM injections in adults, and it states that there are “no data to document the necessity of aspiration” in children.

A different approach to this issue was taken by Ipp *et al.* ^2^ through a survey where the actual practice of end users was evaluated. This survey established that 74% of respondents aspirated prior to IM vaccine administration. However, of these only 3% aspirated for the recommended 5–10 seconds; the remaining applied negative pressure for <5 seconds. The same group went on to conduct a randomized controlled trial in which they compared two injection techniques: the standard approach, which included aspiration for 5–10 seconds, and the pragmatic approach, which excluded aspiration entirely ^50^. They concluded that IM vaccinations using the pragmatic approach were less painful and there were no benefits to following the standard approach. Jablecki ^60^ has suggested a technique for choosing a site for administering IM injection that is relatively pain free by understanding the anatomy of cutaneous innervation at the selected site. This may mitigate the effect of increased pain in the standard approach. Similarly, Philippe Duclos, WHO/Vaccines and Biologicals, has recommended against aspiration prior to injection with a view to minimizing pain ^61^. More recently, a 2007 study of 113 infant vaccinations compared rapid IM injection without aspiration with slow IM injection with aspiration, and found the non-aspiration method to be associated with less pain based on behavioral pain ratings ^41, 62^. Similarly, in 2009, a systematic review of 19 randomized controlled trials involving 2,814 infants and children found that immunization pain can be decreased by performing a rapid IM injection without aspiration ^41^.

In actual practice, AD syringes are recommended worldwide for vaccinations. While this is a small proportion of all injections given worldwide, it is an important component given that the target population is healthy children, and the risks have to be minimized as much as possible. In general, AD syringes do not permit health workers to aspirate blood. This inability to aspirate with AD syringes has generated a heated debate. In theory, some devices like the BD Soloshot allow for limited aspiration ^63^, but this does not meet the recommended criteria for the amount of negative pressure and duration of aspiration.

A summary of the rationale behind the current recommendation of not aspirating during the administration of IM or SC vaccines is given below.

**1. **  Recommended sites for immunizations do not have major blood vessels; hence the risk of accidentally injecting the vaccine into a blood vessel is thought to be minimal ^63^.
**2. **  AD syringes have been used in mass campaigns for IM injections without any reported adverse effects ^7, 63^ or injury from failure to aspirate ^7, 64, 65^. All complications reported in the literature of intra-arterial injection involved penicillin and other medications and not vaccines ^1^. “It is safe to assume that immunization as a class of IM injection poses less risk to the patient” than other medications, particularly antibiotics ^1, 7, 66, 67^. Hence, according to Clements ^7^, “the practice of aspiration during vaccinations is not evidence-based”.
**3. **  Aspiration can result in wastage of vaccine ^64^.
**4. **  Aspiration prolongs the time that the needle is inside the patient hence increasing the pain experienced by the recipient ^50^.
**5. **  Less control is exercised during two-handed aspiration using a conventional syringe, which may lead to local injury. During a one handed vaccination without aspiration, the vaccinator can use the other hand to control the child ^7^.
**6. **  At present, at the public health level, the use of AD syringes represents best practice to protect the health of the public despite the fact AD syringes do not allow aspiration for the recommended 5–10 seconds. The increased risk presented by eliminating aspiration from routine vaccine administration technique can be mitigated to an extent by a thorough understanding of the anatomy and landmarks of recommended injection sites ^66^.

The WHO appreciates that there is not enough evidence to support the exclusion of aspiration ^1, 7^ at present. As a result, “WHO is neither able to support nor offer alternative actions in relation to aspiration undertaken during the administration of vaccines. Until such time as clear evidence becomes available to indicate which method is preferable, vaccinators should make locally appropriate choices ^7^”. In addition, it is suggested that in individual clinical practice using non-AD syringes, aspiration should continue to be a part of the standard technique for IM injection administration ^66^.

The realization that the information available to the WHO may not be comprehensive is reflected in disclaimers that are incorporated in WHO documents/publications. The joint statement on AD syringes in immunization says, “The World Health Organization does not warrant that the information contained in this publication is complete and correct and shall not be liable for any damages incurred as a result of its use”. All WHO publications state, “All reasonable precautions have been taken by the World Health Organization to verify the information contained in this publication. However, the published material is being distributed without warranty of any kind, either expressed or implied. The responsibility for the interpretation and use of the material lies with the reader. In no event shall the World Health Organization be liable for damages arising from its use. The named authors alone are responsible for the views expressed in this publication”.

***Special areas:*** The conventional syringe, primarily designed for injection, is widely used for aspiration. Sibbit *et al.* ^68^ have found the conventional syringe to be unsuited for aspiration during Fine-Needle Aspiration Cytology (FNAC). Robinson *et al.* ^69^ reported a similar experience using conventional syringes for amniocentesis. Aspiration was found to be unreliable in reducing the risk of IV penetration during intraforaminal cervical and lumbosacral epidural steroid injections ^36, 70^. Loss of control during joint aspirations can result in serious complications ^23– 25, 27– 31, 33, 71– 80^, as was noted during other invasive procedures like pericardiocentesis, amniocentesis and thoracocentesis ^77^. Precision is important where critical organs are involved. Improved control was seen with the FDA-approved one-handed reciprocating syringe ^21^.

### Findings of guidelines and recommendations

***Vaccination:*** According to the Red Book ^57^ published by the American Academy of Pediatrics (AAP), there is no need of aspiration before injection of vaccines or toxoids. Similarly, the US CDC guidelines for administration of vaccines ^65^ have clear instructions not to aspirate before injection (for both IM and SC routes), as no large vessels exist in the recommended injection site. No recommendations were found in the Pink Book ^81^ from the CDC in this regard.

None of the documents dealing with immunization (including immunization in practice module 1–11 from the WHO) suggest that aspiration is required before injection of a vaccine ^82^. The WHO Fact Sheet No 231 on Injection Safety, revised in October 2006 ^83^, focuses primarily on injection safety. Technical details, including aspiration, are not touched upon in this document.

***Injection of medication:*** The UK NHS ^84^ and Public Health Agency Canada ^6^ recommend aspiration before IM injection of medication.

Neither the ICN nor the Nursing and Midwifery Council ^50, 51^ (Europe and British Chapters) have made any kind of recommendation in their guidelines on administration of medication. The official website of the WHO’s Uppsala Monitoring Center (UMC) ^85^ does not list any warnings related to aspiration.

National nursing curricula in Nigeria ^86^ and Pakistan ^87^ do not mention aspiration before injection as a necessary step for IM and SC injections. Similarly, the syllabus for MSc Nursing in India did not elaborate on injection technique. Curricula for primary health workers in Nigeria ^40^ and nursing students in Pakistan’s foremost nursing school (Aga Khan University School of Nursing) do advocate aspiration ^38^ before injection. Similarly, the IndiaCLEN Model Injection Center Program advises aspiration prior to IM injection ^88^. None of the curricula mentioned above make any comment on the duration of aspiration.

The United States Pharmacopeia-National Formulary ^89^, British National Formulary ^90^ and Pakistan Pharma Guide ^91^ make no mention of aspiration before injection.

### Review of ISO guidelines

The ISO recommendations 7886 for sterile hypodermic syringes (acquired via personal communication) were reviewed. Relevant sections from parts 1, 3 and 4 are reproduced below. Part 2 relates to syringes for use with power-driven pumps and is therefore beyond the scope of this review.

**Part 1: Sterile hypodermic syringes for single use -** specifies requirements for sterile single-use hypodermic syringes made of plastic materials and intended for the aspiration of fluids or for the injection of fluids immediately after filling.

**Part 3: Auto-disable syringes for fixed dose immunization -** specifies the properties and performance of sterile single-use hypodermic syringes, with or without needle, made of plastic materials and stainless steel and intended for the aspiration of vaccines or for the injection of vaccines immediately after filling. Upon delivering a fixed dose of vaccine, the syringe is automatically rendered unusable.

**Part 4: Syringes with re-use prevention feature -** specifies requirements for sterile single-use hypodermic syringes made of plastic materials with or without needle, and intended for the aspiration of fluids or for the injection of fluids immediately after filling and of design such that the syringe can be rendered unusable after use.

***ISO Section 5.3: Intended use/application:*** The intended use/application shall be categorized as follows:

Type A: single aspiration and injection.

Type B: multiple plunger aspirations prior to the final intended single use.

The term aspiration used in these guidelines indicates the drawing up of the vaccine or medication into the syringe prior to injection. Aspiration as defined in the context of this review is not directly referred to in these guidelines. It appears that withdrawal of the plunger for blood to be visible in the syringe if the needle tip is placed in a vessel, and for this function to be possible at any position of the piston within the graduated range, was considered for inclusion in the ISO guidelines at some point ^92^. However, this is not included in ISO 7886 at all.

### Findings from product inserts

Product inserts for injectable drugs on the Essential Drug List were obtained from over 20 pharmacies across the city of Karachi, Pakistan ^93^.

Each insert was checked and the level of evidence available was categorized as follows:

1. Clearly mentioned on the leaflet to aspirate before injection
2. No mention of aspiration on the leaflet, but advocates a particular route of administration because of the dangers of side effects
3. Suggests to stick to a particular route regardless of outcome of aspiration before injection

Only 3 drugs out of a total of 108 studied had level 1 evidence (bupivacaine, lidocaine and Pneumococcal 7-valent conjugate vaccine). Level-3 evidence was available for only 1 drug (Dactinomycin). The remaining essential drugs gave level-2 evidence Table 2.

**Table 2.** Summary of results based on information given in product inserts.

Generic Name	Trade Name	Level of Evidence	Statement from the leaflet
Dactinomycin	Cosmegen	3	On intravenous administration of Cosmegen, extravasation may occur with or without an accompanying burning or stinging sensation, even if blood returns well on aspiration of the infusion needle. If any signs or symptoms of extravasation have occurred, the injection or infusion should be immediately terminated and restarted in another vein.
Diphtheria, Pertussis, Tetanus (DPT)	Infanrix	2	Do not administer this product subcutaneously or intravenously.
Tetanus toxoid, reduced diphtheria toxoid and acellular pertussis vaccine	Boostrix	2	Special care should be taken to prevent injection into a blood vessel.
Dacarbazine	DTIC-Dome	2	The calculated dose of the resulting solution is drawn into a syringe and administered *only* intravenously.
Erythromycin lactobionate	Erythrocin	2	Must be administered by continuous or intermittent intravenous infusion only. Due to the irritative properties of erythromycin, I.V. push is an unacceptable route of administration.
Hep B vaccine	Engerix-B	2	Do not inject intravenously or intradermally.
MMR	Priorix Vaccine	2	Your doctor/nurse will ensure that Priorix is not injected into the bloodstream.
Promethazine HCL	Phenergon	2	The preferred route of administration for phenergan injection is by deep IM injection. The proper IV administration of the product is well tolerated, but use of this route is not without some hazards. Not for SC administration.
Pneumococcal 7-valent conjugate Vaccine	Prevnar	1	For intramuscular injection only. Do not inject intravenously. After insertion of the needle, aspirate and wait to see if any blood appears in the syringe, which will help avoid inadvertent injection into a vessel. If blood appears, withdraw the needle and prepare for injection at another site.
Progesterone injection	Progesterone	2	For intramuscular use only.
Flupenthixol	Fluanxol	2	Flupenthixol is administered by deep i.m. injection, preferably in the gluteus maximus. Flupenthixol is NOT for i.v. use.
Immune globulin (Human), I.V.	Gamimune ^®^	2	For intramuscular use only.
Bupivacaine HCl	Sensorcaine	1	It is essential that aspiration for blood or cerebrospinal fluid (where applicable) be done prior to injecting any local anesthetic, both the original dose and all subsequent doses, to avoid intravascular or subarachnoid injection. However, a negative aspiration does *not* ensure against an intravascular or subarachnoid injection.
Thiopental Sodium	Pentothal	2	The drug is prepared as a sterile powder and after reconstitution with an appropriate diluent is administered by the intravenous route. Pentothal is administered by the intravenous route only.
Diazepam	Valium Injection	2	For dosage in pediatric patients > 30 days old, see the specific indications below. When intravenous use is indicated, facilities for respiratory assistance should be readily available. *Intramuscular:* Valium Injection should be injected deeply into the muscle. *Intravenous Use:* (See warnings and precautions: *Pediatric Use*.) The solution should be injected slowly, taking at least 1 minute for each 5 mg (1 mL) given. Do not use small veins, such as those on the dorsum of the hand or wrist. Care should be taken to avoid intraarterial administration or extravasation.
Typhoid Vi Polysaccharide vaccine Typhim Vi	Typhim	2	For intramuscular use only. Do NOT inject intravenously.
Purified inactivated rabies vaccine	Verorab	2	Do not inject intravascularly.
Lidocaine	Xylocaine	1 & 3	It is essential that aspiration for blood or cerebrospinal fluid (where applicable) be done prior to injecting any local anesthetics, both the original and all subsequent doses, to avoid intravascular or subarachnoid injection. However, a negative aspiration does not ensure against an intravascular or subarachnoid injection. Local anesthetic solutions containing antimicrobial preservatives (e.g. methylparaben) should not be used for epidural or spinal anesthesia because the safety of these agents has not been established with regards to intrathecal injection, either intentional or accidental.
Rho (D) Immunoglobin (Human)	RHO	2	Do not inject Intravenously. Do not inject neonate.

## Discussion

Aspiration prior to injection is just one part of the process of performing vaccinations, therapeutic injections and diagnostic/therapeutic procedures. The debate over its inclusion as an essential part of recommended techniques has driven this review, and is likely to continue in the absence of findings from randomized controlled trials. In most instances, general clinical or vaccination experiences guide global recommendations for aspiration. In others, anecdotal reports of adverse events form the basis for inclusion or exclusion of aspiration in standard injection techniques. The sheer number of injections given globally in the preventive and therapeutic sectors makes this omission even more surprising. This appraisal of current guidelines and literature has made it clear that the need for aspiration prior to administering an injection is dependent upon multiple factors, as elaborated below.

Injections given for routine immunizations are different from injections for medications. The minimal risk of side effects combined with defined sites for immunization form one basis of the existing recommendations for eliminating aspiration during immunization. The fact that most AD devices currently in use do not allow for aspiration also appears to have been a major factor in the decision to eliminate aspiration as an essential step prior to IM or SC injection of vaccines. We argue that clinical needs should dictate the development of new devices and not the other way around. Relevant recommendations must be evidence-based and ISO guidelines must be modified to reflect evolving needs. This would drive the device industry to meet the criteria laid down based on scientific rationale.

The drug that is being injected has a direct bearing on the decision to aspirate or not to aspirate. If the drugs to be given have potentially fatal consequences in the event of systemic administration (as in the case of immunotherapy), all possible precautions must be taken. This is even more important in cases where the drug is being administered electively by specialist staff. On the other hand, if there are no serious known sequelae to a drug being injected systemically – as in the case of vaccines – an argument can be made not to aspirate, especially since a huge number of immunizations are performed globally by vaccinators and health workers. Product inserts for 104 injectables on the WHO Essential Drug List were reviewed. Of these, only 3 inserts specified that aspiration should be performed prior to injection. Two of these inserts were for local anesthetic agents and the third was for Pneumococcal 7-valent conjugate vaccine. Other product inserts mentioned the importance of injecting into the desired site, but did not specify aspiration as a way of ensuring this. Clearer instructions must be stated if indeed potentially serious complications may occur if a drug or vaccine is inadvertently administered at a site other than that recommended.

As is apparent from ISO 7886 part 4 for curative injection devices, a global shift towards the increasing the use of re-use prevention syringes in the curative sector is imminent. Devices manufactured to meet these criteria incorporate the function of aspiration. Newer devices are coming into the market in order to address the issues of control over the syringe during aspiration and to increase patient safety. One such device recently approved by the US Food and Drug Agency (FDA) is the highly controllable one handed reciprocating procedure syringe ^21^. Specific procedures where aspiration is performed for diagnostic or therapeutic purposes would benefit from newer devices that are custom-designed to aspirate rather than inject.

A systematic approach would be to conduct randomized controlled trials of the device to reach an unbiased conclusion on the benefits and necessity for aspiration using therapeutic re-use prevention syringes and AD syringes for vaccinations; the appropriate duration of aspiration that yields best results also needs to be determined. If such trials deem that aspiration should be part of the recommended therapeutic and vaccination technique, this would act as the driving force for the device industry to develop appropriate tools to meet these requirements.

## Conclusion

There is a shortage of consistent recommendations regarding aspiration before injection in published literature, regulatory guidelines, and medical and nursing school curricula. There is also no central and easily accessible place where one can access and review information and guidelines regarding the procedure. It is therefore important to bring all the evidence, published and otherwise, to the forefront for clinicians, researchers, regulatory bodies and device manufacturers so that they can make an informed decision. Based on our findings, the need for aspiration prior to administering an injection is dependent upon multiple factors. Systemic adverse effects profile and mode of delivery (IV vs IM and SC) of drugs plays a significant role in the decision to aspirate or not to aspirate. There is ample evidence that suggests that aspiration may not be required for IM and SC injections, while for IV injections the systemic side effects of the drug should be considered when aspirating before any injection.

## Literature search terms

“Aspiration”, “injection”, “technique”, “procedure”, “guidelines”, “standards”, “efficacy”, “complications”, “pain”, “trauma”, “administration”, “intramuscular”, “intravascular”, “intradermal”, “subcutaneous”, “syringe”, “auto-disable syringe”, “Z-track”, “immunotherapy”, “epinephrine”, “insulin”, “dental”, “immunization”, “vaccination”, “medication”, “rapid”, “fine-needle”, “pain”, “trauma”.

## References

[1] Kamlesh RL Lala MK: Intramuscular Injection: review and guidelines. *Indian Pediatr.* 2003;40(9):835–845. 14530543

[2] Ipp M Sam J Patricia PC: Needle aspiration and intramuscular vaccination. *Arch Pediatr Adolesc Med.* 2006;160(4):451. 10.1001/archpedi.160.4.451-a 16585496

[3] Mallet J Christopher B: The Royal Marsden NHS Trust Manual of Clinical Nursing Procedure. 4th ed. London: Blackwell Science.1996 Reference Source

[4] Workman B: Safe injection techniques. *Nurs Stand.* 1999;13(39):47–53. 10.7748/ns1999.06.13.39.47.c2623 10497490

[5] Crawford CL Johnson JA: To aspirate or not: an integrative review of the evidence. *Nursing.* 2012;42(3):20–5. 10.1097/01.NURSE.0000411417.91161.87 22343951

[6] Canada, PHAo, Canadian Immunization Guide. 7th ed. Ottawa. Publishing and Depository Services Public Works and Government Services Canada: Ontario.2006 Reference Source

[7] J CC: Aspiration before injection, in SIGN.2003;1–2.

[8] Aceves-Avila FJ Delgadillo-Ruano M Ramos-Remus C: The first descriptions of therapeutic arthrocentesis: a historical note. *Rheumatology (Oxford).* 2003;42(1):180–3. 10.1093/rheumatology/keg001 12509634

[9] Guggi V Calame L Gerster JC: Contribution of digit joint aspiration to the diagnosis of rheumatic diseases. *Joint Bone Spine.* 2002;69(1):58–61. 10.1016/S1297-319X(01)00342-6 11858358

[10] Lane JG Falahee M Wojtys EM: Pyarthrosis of the knee. Treatment considerations. *Clin Orthop Rel Res.* 1990;45(252):198–204. 2302885

[11] Johnson MW: Acute knee effusions: a systematic approach to diagnosis. *Am Fam Physician.* 2000;61(8):2391–400. 10794580

[12] Manadan AM Block JA: Daily needle aspiration versus surgical lavage for the treatment of bacterial septic arthritis in adults. *Am J Ther.* 2004;11(5):412–5. 10.1097/01.mph.0000087296.80768.1e 15356433

[13] Dooley P Martin R: Corticosteroid injections and arthrocentesis. *Can Fam Physician.* 2002;48:285–92. 11889888 PMC2213980

[14] Brown PW: Arthrocentesis for diagnosis and therapy. *Surg Clin North Am.* 1969;49(6):1269–78. 5359829

[15] Lee AH Chin AE Ramanujam T: Gonococcal septic arthritis of the hip. *J Rheumatol.* 1991;18(12):1932–3. 1795336

[16] Kesteris U Wingstrand H Forsberg L: The effect of arthrocentesis in transient synovitis of the hip in the child: a longitudinal sonographic study. *J Pediatr Orthop.* 1996;16(1):24–9. 10.1097/00004694-199601000-00006 8747350

[17] Weidner S Keller W Kellner H: Interventional radiology and the musculoskeletal system. *Best Pract Res Clin Rheumatol.* 2004;18(6):945–56. 10.1016/j.berh.2004.05.011 15501191

[18] Bureau NJ Ali S Chhem RK: Ultrasound of musculoskeletal infections. *Semin Musculoskelet Radiol.* 1998;2(3):299–306. 10.1055/s-2008-1080109 11387109

[19] Grassi W Farina A Filippucci E: Sonographically guided procedures in rheumatology. *Semin Arthritis Rheum.* 2001;30(5):347–53. 10.1053/sarh.2001.19822 11303307

[20] Feldmann H: [2000-year history of the ear syringe and its relationship to the enema. Images from the history of otorhinolaryngology, represented by instruments from the collection of the Ingolstadt Medical History Museum]. *Laryngorhinootologie.* 1999;78(8):462–7. 10.1055/s-2007-996909 10488468

[21] Sibbitt W Jr Sibbitt RR Michael AA: Physician control of needle and syringe during aspiration-injection procedures with the new reciprocating syringe. *J Rheumatol.* 2006;33(4):771–78. 16511940

[22] Draeger HT Twining JM Jhonson CR: A randomised controlled trial of the reciprocating syringe in arthrocentesis. *Ann Rheum Dis.* 2006;65(8):1084–7. 10.1136/ard.2005.045781 16339287 PMC1798237

[23] Roberts WN Hayes CW Breitbach SA: Dry taps and what to do about them: a pictorial essay on failed arthrocentesis of the knee. *Am J Med.* 1996;100(4):461–4. 10.1016/S0002-9343(97)89524-1 8610735

[24] Lobo A Lightman S: Vitreous aspiration needle tap in the diagnosis of intraocular inflammation. *Ophthalmology.* 2003;110(3):595–9. 10.1016/S0161-6420(02)01895-X 12623828

[25] Qublan HS Al-Jader K Al-Kaisi NS: Fine needle aspiration cytology compared with open biopsy histology for the diagnosis of azoospermia. *J Obstet Gynaecol.* 2002;22(5):527–31. 10.1080/0144361021000003690 12521423

[26] Jauniaux E Holmas A Hyett J: Rapid and radical amniodrainage in the treatment of severe twin-twin transfusion syndrome. *Prenat Diagn.* 2001;21(6):471–6. 10.1002/pd.96 11438952

[27] Cederholm M Haglund B Axelsson O: Maternal complications following amniocentesis and chorionic villus sampling for prenatal karyotyping. *BJOG.* 2003;110(4):392–9. 10.1046/j.1471-0528.2003.02091.x 12699801

[28] Farran I Sánchez M Mediano C: Early amniocentesis with the filtration technique: neonatal outcome in 123 singleton pregnancies. *Prenat Diagn.* 2002;22(10):859–63. 10.1002/pd.424 12378565

[29] Papp C Papp Z: Chorionic villus sampling and amniocentesis: what are the risks in current practice? *Curr Opin Obstet Gynecol.* 2003;15(2):159–65. 12634608 10.1097/00001703-200304000-00011

[30] Moore KP Wong F Gines P: The management of ascites in cirrhosis: report on the consensus conference of the International Ascites Club *Hepatology.* 2003;38(1):258–66. 10.1053/jhep.2003.50315 12830009

[31] Murthy SV Hussain ST Gupta S: Pseudoaneurysm of inferior epigastric artery following abdominal paracentesis. *Indian J Gastroenterol.* 2002;21(5):197–8. 12416751

[32] Webster ST Brown KL Lucey MR: Hemorrhagic complications of large volume abdominal paracentesis. *Am J Gastroenterol.* 1996;91(2):366–8. 8607508

[33] Nettleman MD Bock MJ Nelson AP: Impact of procedure-related complications on patient outcome on a general medicine service. *J Gen Intern Med.* 1994;9(2):66–70. 10.1007/BF02600202 8164079

[34] WHO, Global Facts & Figures. A safe injection does not harm the recipient, does not expose the health care worker to any risk and does not result in waste that is dangerous for the community, in *SAFETY OF INJECTIONS* .2006; **33**:4.

[35] Rodger MA King L: Drawing up and administering intramuscular injections: a review of the literature. *J Adv Nurs.* 2000;31(3):574–582. 10.1046/j.1365-2648.2000.01312.x 10718876

[36] Furman MB Giovanniello MT O’Brien EM: Incidence of intravascular penetration in transforaminal cervical epidural steroid injections. *Spine (Phila Pa 1976).* 2003;28(1):21–25. 12544950 10.1097/00007632-200301010-00007

[37] Higgins D: Subcutaneous Injections. *Nursing times.* 2004;100(50):32–3. 15633841

[38] Rozani N: Aga Khan University School of Nursing enrichement program: Skills check list mannual, ed. AHN. Vol. 1. 2007, Karachi. 10.

[39] Clinical Skills: Intramuscular Injections. *Nursing times.* 2003;99(26):27. 12875112

[40] Nigeria C.h.p.r.b.o: Practical assesment record for cummunity health extension workers. Instructors Guide Book.2006.

[41] Taddio A: Physical interventions and injection techniques for reducing injection pain during routine childhood immunizations: systematic review of randomized controlled trials and quasi-randomized controlled trials. *Clin Ther.* 2009;31(Suppl 2):S48–76. 10.1016/j.clinthera.2009.07.024 19781436

[42] Hayes C: Injection technique subcutaneous. *Nursing times.* 1998;94(41):suppl 1–2. 9832866

[43] WHO, I SIGN. Best Infection Control Practices for Skin-Piercing Intradermal, Subcutaneous, and Intramuscular Needle Injections.2001; [cited 2008 June 23]. Reference Source

[44] WHO. A guide for supervising injections. Feb 2004 April 2008 [cited 2013 March 15]; Feb 12. 2004: [1–16]. Reference Source

[45] Waibel KH: Aspiration before immunotherapy injection is not required. *J Allergy Clin Immunol.* 2006;118(2):525–6. 10.1016/j.jaci.2006.04.008 16890784

[46] Miller JD Bell JB Lee RJ: Blood return on aspiration before immunotherapy injection. *J Allergy Clin Immunol.* 2006;119(2):512. 10.1016/j.jaci.2006.09.015 17291866

[47] Guarneri F: Aspiration before subcutaneous immunotherapy injection: Unnecessary or advisable? *J Allergy Clin Immunol.* 2007;119(2):512–513. 10.1016/j.jaci.2006.10.010 17140650

[48] Geller M: Aspiration before immunotherapy injection is required. *J Allergy Clin Immunol.* 2007;120(1):220–1. 10.1016/j.jaci.2007.02.039 17418378

[49] H WK: Reply: Letter to the editor. *J Allergy Clin Immunol.* 2006;120(1).

[50] Council NM: Guidelines for the administration of medicine.2002 [cited 2014 March 15]. Reference Source

[51] Council NM: Standards for medicines management.2004 [cited 2014 March 15]. Reference Source

[52] Corkery PF Barrett BE: Aspiration using local anesthetic catridges with an elastic recoil diaphragm. *J Dent.* 1973;2(2):72–74. 10.1016/S0300-5712(73)80022-3 4533004

[53] Meechana JG Ramacciato JC McCabea JF: A comparison of the aspirating abilities of re-usable and partly disposable dental cartridge syringes *in vitro*. *J Dent.* 2006;34(1):41–47. 10.1016/j.jdent.2005.03.003 15907358

[54] Petersen JK: Efficacy of a self-aspirating syringe. *Int J Oral Maxillofac Surg.* 1987;16(2):241–4. 10.1016/S0901-5027(87)80140-6 3110327

[55] Control, C.o.D. Sample Screening Form Dental Safety Syringes and Needles.2002. [cited 2014 March 15].

[56] Calin MA Parasca SV Savastru R: Optical techniques for the noninvasive diagnosis of skin cancer. *J Cancer Res Clin Oncol.* 2013;139(7):1083–104. 10.1007/s00432-013-1423-3 23552870 PMC11824606

[57] Pickering LK EG V: Ill. Active and Passive Immunization: Report of the Committee on Infectious Diseases. 26th ed. Red book. 2003: American Academy of Pediatrics. Reference Source

[58] Immunization, N.A.C.o., Canadian Immunization Guide. H. Canada, Editor. 2002, Public Health Agency of Canada, Infectious Disease and Emergency Preparedness Branch, Centre for Infectious Disease Prevention and Control: Ontario.38–40. Reference Source

[59] CDC. Vaccine Administration. In: General recommendations on Immunisation. Eds. William LA, L.P., Benjamin S, Bruce W, John lskander, John Watson, Atlanta, USA. MMWR.2002;51(RR02):1–36. Reference Source

[60] Jablecki CK: Letter to the Editor. *Nursing Res.* 2000;49(5):244 Reference Source 10.1097/00006199-200009000-00002 11009118

[61] Duclos P: *WHO/V&B.* SIGN January 2003 July 2008 [cited 2014 March 15].1–2.

[62] Ipp M Taddio A Sam J: Vaccine-related pain: randomised controlled trial of two injection techniques. *Arch Dis Child.* 2007;92(12):1105–8. 10.1136/adc.2007.118695 17686797 PMC2066084

[63] Catlin M Crook B: Giving safe injections: introducing auto-disable syringes. PATH Seattle, WA U.S.A.2000 Reference Source

[64] Program NI: Epidemiology and Prevention of Vaccine-Preventable Diseases.2007; [cited 2014 March 15]. Reference Source

[65] APPENDIX D. Vaccine Administration.2007; [cited 2008 July 1]. Reference Source

[66] Nicoll LH: IM injection: updated information. SIGNpost September.2002;1–2. [cited 2014 March 15].

[67] Nicoll LH Hesby A: Intramuscular injection: an integrative research review and guideline for evidence-based practice. *Appl Nurs Res.* 2002;15(3):149–162. 10.1053/apnr.2002.34142 12173166

[68] Sibbitt RR Sibbitt WL Jr Nunez SE: Control and performance characteristics of eight different suction biopsy devices. *J Vasc Interv Radiol.* 2006;17(10):1657–1669. 10.1097/01.RVI.0000236837.47302.8E 17057008

[69] Robinson JN Loeffler HH Norwitz ER: A syringe adapter to facilitate aspiration at amniocentesis. *Obstet Gynecol.* 2000;96(1):138–140. 10928904 10.1016/s0029-7844(00)00841-3

[70] Furman MB O'Brien EM Zgleszewski TM: Incidence of intravascular penetration in transforaminal lumbosacral epidural steroid injections. *Spine (Phila Pa 1976).* 2000;25(20):2628–2632. 10.1097/00007632-200010150-00014 11034648

[71] Yankelevitz DF Hayt D Henschke CI: Transthorasic needle biopsy. What size syrine? *Clin Imaging.* 1995;19(3):208–209. 10.1016/0899-7071(94)00070-S 7553439

[72] Castellote J Xiol X Cortés-Beut R: Complications of thoracentesis in cirrhotic patients with pleural effusion. *Rev Esp Enferm Dig.* 2001;93(9):566–75. 11767433

[73] Doyle JJ Hnatiuk OW Torrington KG: Necessity of routine chest roentgenography after thoracentesis. *Ann Intern Med.* 1996;124(9):816–20. 10.7326/0003-4819-124-9-199605010-00005 8610950

[74] Sassoon CS Light RW O'Hara VS: Iatrogenic pneumothorax: etiology and morbidity. Results of a Department of Veterans Affairs Cooperative Study. *Respiration.* 1992;59(4):215–20. 10.1159/000196061 1485006

[75] Callahan JA Seward J: Pericardiocentesis Guided by Two-Dimensional Echocardiography. *Echocardiography.* 1997;14(5):497–504. 10.1111/j.1540-8175.1997.tb00757.x 11174988

[76] Bastian A Meissner A Lins M: Pericardiocentesis: differential aspects of a common procedure. *Intensive Care Med.* 2000;26(5):572–6. 10.1007/s001340051206 10923732

[77] Salem K Mulji A Lonn E: Echocardiographically guided pericardiocentesis - the gold standard for the management of pericardial effusion and cardiac tamponade. *Can J Cardiol.* 1999;15(11):1251–5. 10579740

[78] Tsang TS Barnes ME Gersh BJ: Outcomes of clinically significant idiopathic pericardial effusion requiring intervention. * Am J Cardiol.* 2003;91(6):704–7. 10.1016/S0002-9149(02)03408-2 12633802

[79] Tsang TS El-Najdawi EK Seward JB: Percutaneous echocardiographically guided pericardiocentesis in pediatric patients: evaluation of safety and efficacy. *J Am Soc Echocardiogr.* 1998;11(11):1072–7. 9812101 10.1016/s0894-7317(98)70159-2

[80] Duvernoy OBJ Borowiec J Helmius G: Complications of percutaneous pericardiocentesis under fluoroscopic guidance. *Acta Radiol.* 1992;33(4):309–13. 10.1080/02841859209173184 1633040

[81] Atkinson W Hamborsky J McIntyre L: The Pink Book. 10th ed. 2nd printing ed. Epidemiology and Prevention of Vaccine-Preventable Diseases. Washington DC: Public Health Foundation: Centers for Disease Control and Prevention.2008 Reference Source

[82] Kaic B: Aspiration before injection. SIGNpost January 2003 July 2008. Reference Source

[83] WHO. Fact sheet N°231 Injection safety.2002 [cited 2008 March 21]. Reference Source

[84] NHS. Administration of medicines through intramuscular injections - Guidelines.2006 [cited 2014 March 15]. Reference Source

[85] Center, T.U.M. Adverse reactions newsletter.1996 Reference Source

[86] Nigeria, C.h.p.r.b.o., Curriculum for higher diploma in cummunity health. Instructors Guide Book.2006.

[87] Curriculum, P.N.C.B.N.1990;50–51.

[88] IndiaCLEN. Model Injection Centres (MICs): A Program to Improve Injection Practices in the Country (2005–2006). Reference Source

[89] Nguyen QD Tatlipinar S Shah SM: Vascular endothelial growth factor is a critical stimulus for diabetic macular edema. *Am J Ophthalmol.* 2006;142(6):961–9. 10.1016/j.ajo.2006.06.068 17046701

[90] British National Formulary.2007; [cited 2014 March 15]. Reference Source

[91] Pakistan Pharma Guide. [cited 2014 March 15]. Reference Source

[92] Battersby A: “To aspirate or not to aspirate” that is the question. SIGN January 2003 July 2008 [cited 2014 March 15]; July 2008: [1–2]. Reference Source

[93] Safety, C.o.I. Supplementary Material. 2009 [cited 2014 March 15]. Reference Source

