# Supplementary material for: Aspiration in injections: should we continue or abandon the practice?
Source: F1000Res. 2017 Mar 1;3:157. Originally published 2014 Jul 10. [Version 3] doi: 10.12688/f1000research.1113.3 (PMC5333604; doi:10.12688/f1000research.1113.3)
Supplement: Peer review history

# Peer review: Referee response for version 3

**DOI:** 10.5256/f1000research.11844.r20641

**Published:** 2017-03-01

**Authors:** Cees Smit Sibinga

**Affiliations:** 1 IQM Consulting for International Development of Quality Management in Transfusion Medicine, Zuidhorn, Netherlands

Thanks for the improved conclusion with some useful directions. That completes this interesting and disputed review on the issue of aspiration in injections. Congratulations.

I have read this submission. I believe that I have an appropriate level of expertise to confirm that it is of an acceptable scientific standard.

# Peer review: Referee response for version 2

**DOI:** 10.5256/f1000research.11551.r19832

**Published:** 2017-02-06

**Authors:** Cees Smit Sibinga

**Affiliations:** 1 IQM Consulting for International Development of Quality Management in Transfusion Medicine, Zuidhorn, Netherlands

The manuscript has certainly improved.

 However, the added conclusion is too weak and should be enforced by including the conclusive considerations regarding the use of AD syringes, and a recommendation to the international world to synchronize guidelines and teaching materials on the practice of injection with or without aspiration.

 The recommendation that 'further studies looking at the need for aspiration during injection should be conducted' is a crude generality and might follow a short listing of practical/feasible recommendations. Afterall the manuscript is written as a review, not a reporting of original research on the question 'to aspirate or not' when injecting a substance.

 Minor comments:

 Start the current conclusion with the word 'There' instead of 'The'.

 The typo in the abstract mentioned has not yet been corrected - '... medical/-nursing ..' = '... medical/nursing ..' (delete the dash!)

I have read this submission. I believe that I have an appropriate level of expertise to confirm that it is of an acceptable scientific standard, however I have significant reservations, as outlined above.

# Peer review: Referee response for version 1

**DOI:** 10.5256/f1000research.1271.r11456

**Published:** 2015-12-30

**Authors:** Ankit Balani, Sandhya Sarjare

**Affiliations:** 1 Department of Radiology, Yashoda Hospitals, Secunderabad, India; 2 Department of Radiology, Yashoda Hospital, Secunderabad, India

The manuscript is intelligently written and authors have provided a literature review of need of pre-injection aspiration and discussed its utility in clinical practice. However, we would like to make a few pertinent observations:

The authors have not concluded the review article and it would be appreciated if they could summarize their observations from the literature review and provide an appropriate conclusion giving the readers an insight into the necessity of pre-injection aspiration.

Grammatical error in abstract: **medical/-nursing schools**... needs to be replaced with **medical/nursing**.

Grammatical error in literature review (methodology) - first line - **An** literature review to be replaced by **A** literature review.

Grammatical error in Results - Immunization - Page 5: AD syringes do not permit health workers to aspirate **for** blood to be replaced **to aspirate blood**. 

Grammatical error in Findings of guidelines and recommendation (injection of medication) - Page 6 - ..Msc Nursing in India **does not elaborated on **injection techniques needs to be replaced with **did not elaborate** injection techniques.

In review of ISO guidelines, ISO section 5.3 - Page 6 - ...vaccine or medication into the syringe prior to **aspiration** to be replaced by ....prior to **injection**.  

Spelling errors in Table 2 including Diphtheria and Tetanus in second row need to be rectified.

We have read this submission. We believe that we have an appropriate level of expertise to confirm that it is of an acceptable scientific standard.

# Peer review: Referee response for version 1

**DOI:** 10.5256/f1000research.1271.r5402

**Published:** 2014-09-04

**Authors:** Cees Smit Sibinga

**Affiliations:** 1 IQM Consulting for International Development of Quality Management in Transfusion Medicine, Zuidhorn, Netherlands

The authors have compiled important information based on both literature review and the analysis of guidelines and medication inserts on the issue of pre-injection aspiration once a needle is inserted in or under the skin.

However, the review might be improved by bringing in a more systematic approach in which the results follow the description in the Methodology. That would lead to a consistent and orderly review of the different aspects identified. It is also recommended to include in the introduction a definition of aspiration in the context of the review.

Table 2 shows several spelling errors that need to be corrected.

On page 6/11 (second column, last paragraph) the sentence ‘…. *the drawing up of the vaccine or medication into the syringe prior to aspiration*.’ erroneously uses the word *aspiration* where this should be *injection.*

A clear conclusion with feasible recommendations to come to evidence pro or con, or at least a nuancing in the pro and con of pre-injection aspiration would certainly contribute, as this is not really expressed.

I have read this submission. I believe that I have an appropriate level of expertise to confirm that it is of an acceptable scientific standard, however I have significant reservations, as outlined above.
